# Analysis of Influences of Sjögren’s Disease and Anti-Ro/SS-A Antibodies on Clinical Course of Patients with Rheumatoid Arthritis Complicated by Lymphoproliferative Disorders: A Pilot Study

**DOI:** 10.3390/jcm15062271

**Published:** 2026-03-17

**Authors:** Yoshiro Horai, Shota Kurushima, Hideki Nakamura, Atsushi Kawakami

**Affiliations:** 1 Department of Rheumatology, Sasebo City General Hospital, 9-3 Hirase-Cho, Sasebo 857-8511, Japan; 2 Department of Immunology and Rheumatology, Division of Advanced Preventive Medical Sciences, Nagasaki University Graduate School of Biomedical Sciences, Nagasaki 852-8523, Japan; 3 Division of Hematology and Rheumatology, Department of Medicine, Nihon University School of Medicine, Tokyo 173-8610, Japan

**Keywords:** anti-Ro/SS-A antibodies, lymphoproliferative disorders, methotrexate, rheumatoid arthritis, Sjögren’s disease, tocilizumab

## Abstract

**Background/Objectives**: Lymphoproliferative disorders (LPDs) are adverse effects of methotrexate (MTX) prescribed for rheumatoid arthritis (RA). Sjögren’s disease (SjD), for which the presence of anti-Ro/SS-A antibodies (Abs) is a diagnostic criterion, might accompany RA and be a risk factor for LPDs. We conducted a retrospective study to analyze the effects of SjD or anti-Ro/SS-A Ab positivity on the clinical course of patients with RA complicated by LPDs. **Methods**: We retrospectively analyzed 25 patients in our department who had RA complicated by LPDs, specifically collecting clinical information regarding the complications of SjD and positivity for anti-Ro/SS-A Abs. **Results**: In total, 25 patients with RA were included in this study, 3 of which were diagnosed with SjD by attending physicians based on sicca symptoms and positiveness of anti-Ro/SS-A antibodies. No significant differences in clinical characteristics except for SjD diagnosis given by attending physicians were found between the patients positive for anti-Ro/SS-A Abs and the patients negative for anti-Ro/SS-A Ab. The most common histologic LPD subtype was diffuse large B cell lymphoma, while mucosa-associated lymphoid tissue lymphoma, the histologic subtype often diagnosed as SjD-LPD, was found in only one patient, who was positive for anti-Ro/SS-A Abs without an SjD diagnosis. There were no significant differences in the intervals between the RA and LPD diagnoses and those of SjD and anti-Ro/SS-A Ab positivity. **Conclusions**: While the rate of anti-Ro/SS-A Ab positivity in the study population seemed to be higher than that in the general RA population, any potential effects of SjD on RA-LPD development were not ascertained in this study.

## 1. Introduction

Rheumatoid arthritis is a systemic rheumatic disease characterized by synovitis—mainly affecting small joints—and the presence of autoantibodies, such as rheumatoid factor and anti-citrullinated protein antibodies, and extraarticular complications, such as interstitial pneumonia and cardiovascular disease [1]. Prior to the development of disease-modifying antirheumatic drugs (DMARDs), many patients with RA suffered from advanced joint damage resulting in decreased ability to perform activities of daily living. As therapy has been revolutionized through DMARDs, the therapeutic goal for patients with RA is not only soothing arthralgia but also preventing joint destruction [2]. Considering this, in the 2022 European Alliance of Associations for Rheumatology (EULAR) recommendations for RA, it is stated that DMARDs should be initiated as soon as the diagnosis is given [3]. Even in the era of molecular targeted therapy, which has enabled the superior effectiveness of RA treatments including biological DMARDs (bDMARDs) and Janus kinase inhibitors (JAKis), methotrexate (MTX) is still positioned as the anchor drug for RA based on its promising efficacy and the adjuncts required for other DMARDs such as infliximab, a type of tumor necrosis factor inhibitor (TNFi) [4]. MTX is still considered the first-line therapy for RA according to the 2022 EULAR recommendations [3], as well as the updated guidance provided by Japan College of Rheumatology (JCR) [5]. However, lymphoproliferative disorders (LPDs) are well-known to be life-threatening adverse effects of MTX use [6]. Several DMARDs other than MTX are also presumed to be associated with LPDs; for example, LPDs associated with immunosuppressants are referred to as other iatrogenic immunodeficiency-associated LPDs [2]. Although spontaneous regression after MTX withdrawal can be expected in more than a few cases [7], chemotherapy is required for the remaining cases. Treating RA after LPD-related complications often consists of immunomodulator and glucocorticoid (GC) DMARDs. However, treatment without MTX is often insufficient to achieve remission. At the same time, insufficient control of RA leads to deteriorated activities of daily living in patients, and it is presumed that high-disease-activity RA might also contribute to LPD development [8]. Treating RA requires a balance between achieving sufficient disease control and avoiding adverse effects, including LPDs.

Sjögren’s disease (SjD), recently renamed from Sjögren’s syndrome, is generally considered a rheumatic disease frequently characterized by sicca symptoms affecting the mouth and eyes. However, people with SjD often exhibit extraglandular symptoms including arthritis, making it difficult to differentiate the condition from RA, and organ-related symptoms such as interstitial pneumonia, which might affect prognosis [9,10]. Furthermore, SjD is considered a risk factor for LPDs [10], similarly to RA. Analyzing the synergetic risks contributing to LPD development in RA, complicating SjD, would be important for the appropriate selection of DMARDs for the condition. Anti-Ro/SS-A antibodies (Abs) are serum diagnostic markers for SjD, and have been adopted as such in globally accepted classification criteria for SjD such as the 2002 American–European Consensus Group (AECG) Classification Criteria [11] and the American College of Rheumatology (ACR)/EULAR 2016 classification criteria [12]. Anti-Ro/SS-A Ab positivity is also found in a certain proportion of patients with RA: Matsudaira et al. previously reported that 3–15% of patients with RA were positive for anti-Ro/SS-A Abs [13]. The therapeutic effects of several DMARD classes were reported to be decreased in patients with SjD or anti-Ro/SS-A Ab positivity [8]. Taken together, it seems important to analyze the influences of SjD pathophysiology on the therapeutic responses of DMARDs and the risks of LPDs in patients with RA. However, there are currently no studies on SjD’s possible contribution to the development of LPDs associated with RA and/or MTX [14].

In this study, we retrospectively analyzed the effects of SjD and/or anti-Ro/SS-A Ab positivity on the clinical course of patients with RA complicated by LPDs.

## 2. Patients and Methods

### 2.1. Patients and Clinical Items

We retrospectively analyzed patients who visited our department from April 2016 to March 2024 and were diagnosed with RA complicated by LPDs. The patients who met the inclusion criteria had visited our hospital with diagnoses of RA and LPDs, which were recorded in the Japanese health insurance system. Specifically, we collected clinical information regarding SjD and anti-Ro/SS-A antibody positivity, the latter measured using a chemiluminescent enzyme immunoassay from a commercial company external to our hospital, following their protocol, or using results collected from referral letters sent by neighboring hospitals. As well as information on SjD and anti-Ro/SS-A antibodies, we also collected clinical data such as the patient’s age at LPD onset, the patient’s sex, the interval between RA and LPD diagnoses, the dose and duration of MTX administration, the administrated dose of GC, prescription of DMARDs other than MTX, histologic subtype of LPD, and mortality. We excluded patients for whom clinical information crucial for the aims of the study, such as the clinical course of RA and histologic subtype of LPD, was not obtained. The study algorithm was as follows: 1. Extraction of patients who were eligible for the study, i.e., those with a diagnosis of RA complicating an LPD. 2. Exclusion of patients who met the exclusion criteria. 3. Collection of the above-mentioned clinical information for each patient from medical records. 4. Comparison of patients with and without SjD and with or without positive anti-Ro/SS-A Abs. The protocol for this retrospective study was approved by the Institutional Review Board of Sasebo City General Hospital (no. 2024-A019; approval date: 24 June 2024). The analysis was only conducted at Sasebo City General Hospital. This study’s retrospective and single-center design is regarded as a limitation.

### 2.2. Diagnosis

RA was diagnosed according to the 2010 ACR/EULAR classification criteria [15], based on history taking, physical examinations performed by attending physicians from our department, and laboratory findings. SjD was diagnosed by JCR-board-certified rheumatologists based on sicca symptoms, decreased saliva/tear secretion, and anti-Ro/SS-A Abs positivity, referring to the 2002 AECG Classification Criteria [11] and the ACR/EULAR 2016 classification criteria [12].

### 2.3. Statistical Analysis

We used the χ^2^ test, Student’s *t* test, or Mann–Whitney *U*-test for detecting any differences in results between the group with positivity for anti-Ro/SS-A Abs and the group negative for anti-Ro/SS-A Abs. Values of *p* < 0.05 were considered significant. Statistical analysis was performed using IBM SPSS Statistics 30.

## 3. Results

**Figure 1:**
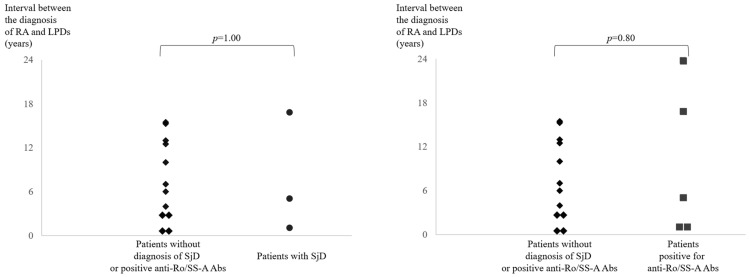
Intervals between the diagnoses of RA and LPDs in patients without diagnosis of SjD and negative for anti-Ro/SS-A Abs compared to those in patients with SjD and patients positive for anti-Ro/SS-A Abs. Abs, antibodies; LPDs, lymphoproliferative disorders; RA, rheumatoid arthritis; SjD, Sjögren’s disease. All the patients with SjD were positive for anti-Ro/SS-A Abs, while there were two patients positive for anti-Ro/SS-A Abs without diagnosis of SjD.

A total of 25 patients with RA (13 women and 12 men) were included in this study. The mean interval between diagnoses of RA and LPDs was 5.8 years. Almost all patients (96%) had received MTX, with a mean dose and administration period at LPD diagnosis of 8 mg/week and 3.8 years, respectively. Around half of the patients (48%) received GC concomitantly, in whom the median dose of prednisolone (PSL) was 5 mg/day. Three patients were diagnosed as having SjD by attending physicians who gave their expert opinion. Of the 17 patients who were tested for anti-Ro/SS-A Abs, 5 (29%) were found to be positive, which was a higher rate than that previously reported for patients with RA [13]. The most common LPD histologic subtype was diffuse large B cell lymphoma (DLBCL). In contrast, mucosa-associated lymphoid tissue lymphoma, which is the most common histologic subtype of SjD-associated LPD [16], but can also be present in RA-LPD [2], was found in only one patient, who was positive for anti-Ro/SS-A Abs without an SjD diagnosis. Nine patients were positive for Epstein–Barr virus-encoded RNA (EBER). As a treatment for RA after LPD development, GC was prescribed for more than half of the patients (14/25, 56%). Other than GC, immunomodulators such as hydroxychloroquine (HCQ), salazosulfapyridine, iguratimod, and bucillamine were prescribed, and tocilizumab (TCZ) and denosumab were utilized for some patients. HCQ, which is currently indicated on-label for neither RA nor SjD by the Japanese Ministry of Health, Labour, and Welfare (JMHLW), was prescribed for associated SLE. The deaths of eight patients were reported in the medical records (Table 1). We selected patients in whom anti-Ro/SS-A Abs were measured and compared the clinical characteristics distinguishing the patient group positive for anti-Ro/SS-A Abs from the group negative for anti-Ro/SS-A Abs. No statistically significant differences in clinical characteristics except for SjD diagnosis given by attending physicians were found between the two groups (Table 2). There was no significant difference in the intervals between the RA and LPD diagnoses between the patients who were not diagnosed with SjD and negative for anti-Ro/SS-A Abs and the patients with SjD, as is the case regarding the comparison of the patients who were not diagnosed with SjD and negative for anti-Ro/SS-A Abs with the patients who were positive for anti-Ro/SS-A Abs (Figure 1).

TCZ was prescribed for five patients after LPD diagnosis, one of whom continued subcutaneous TCZ even after LPD diagnosis. There were no patients diagnosed as having SjD by the attending physicians, whereas one patient was positive for anti-Ro/SS-A Abs. The histologic subtype of LPD was DLBCL in four patients, while the remaining patient was diagnosed with Hodgkin’s lymphoma. One patient was positive for EBER. Three patients received chemotherapy, including rituximab (RTX), for LPDs, while watchful waiting after MTX withdrawal was offered to the remaining two patients. Except the 80-year old-male patient treated with TCZ and low-dose PSL, all the other cases received MTX-LPD. Subcutaneous TCZ monotherapy was given to one patient, while TCZ was prescribed in combination with low-dose PSL and/or immunomodulators for the other patients. Three patients, including one who had started TCZ before LPD development, continued TCZ without adverse effects, whereas the other two patients discontinued TCZ: one patient passed away due to pulmonary Mycobacterium avium complex infection, and one patient experienced subsequent complicated colitis and splenic abscess. In the former patient, the diagnosis of infection was made two months after the introduction of TCZ. At the introduction of TCZ, their lymphocyte count was around 1200/µL. In the latter patient, the infection was diagnosed two and half years after the introduction of TCZ. At the time TCZ was introduced, their lymphocyte count was around 820/µL.

## 4. Discussion

Patients with RA are considered to be at a high risk of malignancy. In a recent survey of Japanese hospitals, including more than 50 facilities belonging to the National Hospital Organization (NHO), lymphoma was found to be the second most frequent malignancy in patients with RA, following lung cancer and followed by colorectal cancer. While the overall incidence of malignancy was comparable to that of the general population, the frequency of lymphoma, as well as skin cancer, was found to be greater. In this analysis, there was a tendency for lymphoma occurrence to be associated with MTX usage, and statistically significant decreases in lymphoma were found in patients treated with bDMARDs other than TNFis [17]. Whereas SR after MTX withdrawal can be expected in more than a few cases of MTX-LPD, the prognosis in patients with LPDs associated with RA is not necessarily good. In a multicenter study conducted in Japan, the 5-year survival and relapse rates after the spontaneous regression of LPDs in patients with RA were 78.2% and 24.9%, respectively [18]. According to previous studies, LPDs often develop in patients with RA who are aged 60 years or older and have a disease duration exceeding ten years [2]. In this study, the mean age of LPD development was relatively older, and the duration of RA before LPD development was relatively shorter. This might be partly attributable to the current increasing trend in the rate of late-onset RA [19]. The most common LPD histological subtype in this study was DLBCL, which is comparable to findings of previous studies on RA-LPDs [2] and MTX-LPD [20]. However, mucosa-associated lymphoid tissue lymphoma was found in only one patient, who was positive for anti-Ro/SS-A Abs without an SjD diagnosis, and was not found in patients with an SjD diagnosis. Although it is necessary to consider limitations such as the high administration rate of MTX and other DMARDs, the analysis of LPD histological subtypes and intervals between RA and LPD diagnoses in this study suggests that SjD could have a limited effect on RA-LPD development. DLBCL remained dominant, even among anti-Ro/SS-A-positive patients. This may have partially been due to the characteristics of anti-Ro/SS-A Abs; as noted in the Introduction, anti-Ro/SS-A Abs are positive at a certain rate in patients with isolated RA. Therefore, in patients with RA positive for anti-Ro/SS-A Abs without a diagnosis of SjD, LPDs might have a distinct etiology from SjD.

A standard therapeutic strategy for RA after LPD complications is yet to be established. MTX is regarded as the anchor drug for RA, as described above, but it should be avoided after LPD development [2]. TCZ is a bDMARD classified as a non-TNFi; it is an inhibitor of interleukin-6 that activates a signaling pathway associated with tumorigenesis and is also a JAK2/signal transducer and an activator of the transcription 3 pathway [21]. While TCZ showed better clinical efficacy when administered in combination with MTX, TCZ monotherapy was found to be adequately effective in a multicenter clinical trial conducted in Japan [22]. The potential of TCZ to replace MTX after achieving RA remission was shown in another clinical trial conducted in hospitals in Japan [23]. The effectiveness of TCZ even without MTX might be beneficial in patients with RA as this would decrease the risk of LPDs. TCZ was recently reported as a useful RA treatment option after LPD development in a multicenter clinical study including 34 facilities in Japan belonging to the NHO: TCZ administration was associated with lower relapse rates of LPDs in RA treatment after LPD development [24]. In previous studies, benefits of TCZ over other DMARDs were reported for RA with SjD and/or anti-Ro/SS-A Ab positivity: the rate of TCZ continuation was superior to that of TNFis, and it was suggested that the effectiveness of MTX, TNFis, and abatacept was decreased in patients with RA positive for anti-Ro/SS-A Abs, but not of TCZ [8]. However, bDMARDs are associated with increased occurrence rates of opportunistic infections, and TCZ is no exception. Severe infections resulting in TCZ discontinuation were also found in two patients included in the present study. It should be noted that these two patients continued low-dose PSL after TCZ introduction. GC use is associated with adverse events, including infection, in RA patients [25], and it is noted that the lowest dose should be used and for the shortest duration in the above-mentioned drug treatment algorithm published by JCR [26]. This study’s results reiterate the importance of decreasing and terminating GC use after achieving remission with TCZ administration.

The effectiveness and safety of JAKis for RA after LPD development are not clarified. In this study, one patient who was prescribed filgotinib at LPD development was included, and no patients received JAKis after LPD diagnosis. The only patient who had a history of JAKi use achieved both LPD and RA remission after chemotherapy that included RTX. However, monitoring might be required for patients with LPDs after JAKi administration: Harada et al. analyzed MTX-LPD cases to clarify their clinical outcomes and found that prior administration of JAKis was associated with aggressive disease course [27].

This study has several limitations, the most important of which is that it was a retrospective study that used data from a single institution with a limited inclusion period, resulting in a small number of patients, both as a whole and those with SjD. Due to the rare nature of this disease combination, it was difficult to find a large number of patients exhibiting it. Despite this problem, the study is still significant due to the serious consequences for patients with RA. We searched for patients with RA complicating LPDs, and not all patients were tested for SjD by attending physicians because those patients were referred as possible RA, not SjD. This resulted in missing anti-Ro/SS-A Ab data for eight patients, which affected the positive rate reported in this study. The missing rate of testing anti-Ro/SS-A Ab is not low, which would be an element bringing selection bias in this study, and should be overcome by multi-center studies with more patients, as mentioned later. Only Japanese patients were included in the study population; therefore, these results are not applicable to the global population. However, there are international differences in SjD epidemiology and clinical practice [9]; thus, an investigation specific to each country would also be important as well as studies based on big data collected globally. As described above, the JMHLW does not approve the prescription of HCQ for RA, but it is considered an option for patients with a high-risk profile and as part of multi-drug therapy in the ACR’s 2021 treatment guidelines [28]. Outside Japan, RTX is a therapeutic option for patients with RA after LPD development; however, RA is not an on-label indication for RTX according to the JMHLW as of the writing of this article. However, patients who receive chemotherapy comprising RTX for LPDs often maintain RA remission for a certain period, as seen in some of the patients included in this study. The effectiveness of a 12-month RTX course for RA after LPD development was shown in a retrospective study of a Japanese population [29], and in the JCR’s updated 2024 guidelines, RTX was referred to as a treatment option for RA after LPD development; thus, it could be approved for this use even in Japan [30].

## 5. Conclusions

In this study, the rate of anti-Ro/SS-A Ab positivity in patients with RA seemed to be relatively higher than that in the general RA population; however, no significant differences in clinical characteristics except for SjD diagnosis given by attending physicians were observed between the patients positive for anti-Ro/SS-A Abs and patients negative for anti-Ro/SS-A Abs. As mentioned above, the patient numbers were too small to generalize the conclusions. Conducting multi-center studies with more participants would reveal the features of RA-LPDs in association with SjD and anti-Ro/SS-A Abs in greater detail, and solve the problems arising from small patient numbers and the limited testing of anti-Ro/SS-A Abs found in this study. TCZ was considered an effective option in this study population; however, attending physicians should be cautious in cases of severe infection.

## Figures and Tables

**Table 1:** Clinical features of patients.

Age at the onset of LPD, years	69 (median, 58–80)
Female, *n*	13 (52%)
Interval between the diagnosis of RA and LPDs, years	5.8 (median, 0.5–30)
Administration of MTX, *n*	24 (96%)
Dose, mg/week	8 (median, 4–14)
Duration, years	3.8 (median, 0–15.5 *)
Administration of GC, *n*	12 (48%)
Dose equivalent to PSL, mg/day	5 (median, 1–10)
DMARDs other than MTX, *n*	SASP 5, IGU 5, TAC 2, BUC 2, TCZ 1, JAKis 2 (FIL 1, unknown subclass 1)
Diagnosis of SjD, *n*	3 (12%)
Anti Ro/SS–A Ab–positive, *n*	3 (29% **)
Histologic subtype of LPDs, *n*	DLBCL 14, Hodgkin lymphoma 5,
	FL 1, MALT lymphoma 1, Suspicious B-cell lymphoma1, EBV positive mucocutaneous ulcer 1,
	Peripheral T-cell lymphoma 1, Lymphoplasmacytic lymphoma 1
EBER–positive, *n*	9 (56% ***)
Treatment for RA after complicating LPDs, *n*	GC 14, SASP 6, TCZ 5, BUC 3,
	IGU 2, DEN 2, HCQ 1 ****, no treatment 4
Death, *n*	8 (32%)

BUC, bucillamine; DEN, denosumab; DMARDs, disease-modifying antirheumatic drugs; DLBCL, diffuse large B cell lymphoma; EBV, Epstein–Barr virus; EBER, Epstein–Barr virus-encoded RNA; FIL, filgotinib; FL, follicular lymphoma; GC, glucocorticoid; HCQ, hydroxychloroquine; IGU, iguratimod; JAKis, Janus kinase inhibitors; LPDs, lymphoproliferative disorders; MALT, mucosa-associated lymphoid tissue; MTX, methotrexate; PSL, prednisolone; RA, rheumatoid arthritis; SASP, salazosulfapyridine; SjD, Sjögren’s disease; TAC, tacrolimus; TCZ, tocilizumab. * results were unknown in four patients; ** in 17 patients for whom results were obtained; *** in 16 patients for whom results were obtained; **** administered for concomitant systemic lupus erythematosus.

**Table 2:** Comparison of clinical characteristics between two patient groups: with and without anti-Ro/SS-A Abs.

	Anti–Ro/SS–A Abs		
	Positive (*n* = 5)	Negative (*n* = 12)	*p*
Age at the onset of LPD, years	65 (median, 58–80)	70 (median, 63–77)	0.38
Female, *n*	3 (60%)	6 (50%)	1.00
Interval between the diagnosis of RA and LPDs, years	5 (median, 1–23.8)	6.5 (median, 0.5–15.5)	0.80
Administration of MTX, *n*	4 (80%)	12 (100%)	0.64
Dose, mg/week	8 (median, 4–12)	8 (median, 6–10)	0.95
Duration, years	7.9 (median, 0–12)	4.8 (median, 0.5–15.5)	0.77
Administration of GC, *n*	3 (60%)	4 (33%)	0.63
Dose equivalent to PSL, mg/day	5 (median, 3–7)	5 (median, 1–10)	0.92
Diagnosis of SjD, *n*	3 (60%)	0 (0%)	0.02
EBER–positive, *n*	2 (50%) *	2 (33%) **	1.00
Death, *n*	1 (20%)	4 (33%)	1.00

Abs, antibodies; EBER, Epstein–Barr virus-encoded RNA; GC, glucocorticoid; LPDs, lymphoproliferative disorders; MTX, methotrexate; PSL, prednisolone; RA, rheumatoid arthritis; SjD, Sjögren’s disease. * in four patients for whom results were obtained; ** in six patients for whom results were obtained.

## Data Availability

The data underlying this article will be shared on reasonable request to the corresponding author.
